# Telemedicine-guided management of iodinated contrast media-associated anaphylaxis: A case report

**DOI:** 10.1016/j.radcr.2026.07.082

**Published:** 2026-08-22

**Authors:** Ravi V. Soni, Samuel B. Beger, William Ji

**Affiliations:** a Department of Emergency Medicine, John P. and Katherine G. McGovern Medical School, University of Texas Health Science Center at Houston, Houston, TX, USA; b Department of Radiology, Stanford University, Stanford, CA USA; c Uniformed Services University of the Health Sciences, Bethesda, MD, USA

**Keywords:** Iodinated contrast media, Anaphylaxis, Telemedicine, Virtual contrast supervision, Case report

## Abstract

Immediate hypersensitivity reactions to iodinated contrast media (ICM) are uncommon, and ICM-associated anaphylaxis with airway compromise is rare, but time critical. In recent years, radiology has increasingly incorporated telemedicine-enabled workflows, including forms of virtual direct supervision, supported in part by evolving U.S. reimbursement and supervision policies. As of January 1, 2026, the Centers for Medicare and Medicaid Services (CMS) has finalized policies indefinitely permitting direct supervision via real-time 2-way audiovisual communications in specified contexts, such as diagnostic imaging. A 63-year-old woman undergoing outpatient CT pelvis with contrast developed acute urticaria, hypotension, hypoxemia, and upper-airway angioedema within minutes of injection, meeting clinical criteria for anaphylaxis. An on-site technologist initiated emergency protocol and activated a telemedicine emergency alert, prompting real-time audiovisual communication with an already connected remote supervising physician. Under remote physician guidance, the on-site team administered two 0.3 mg intramuscular doses of epinephrine, supplemental oxygen, and airway positioning maneuvers, with subsequent improvement in oxygenation and systolic blood pressure before EMS arrival. The patient was later intubated in the emergency department for airway protection, extubated the following day, and discharged home without neurologic sequelae. This case suggests that telemedicine-enabled supervision platforms may support timely recognition and management of ICM-associated anaphylaxis when coupled with on-site preparedness, trained personnel, and readily available emergency medications and airway adjuncts. However, remotely witnessed emergencies introduce distinct operational risks (eg, connection latency, communication handoffs), and warrant rigorous protocols, simulation-based training, and systems-level redundancy. Similar events may become increasingly relevant as distributed imaging care models expand.

## Introduction

Iodinated contrast media (ICM) are widely used in diagnostic imaging and administered in more than 120 million imaging procedures worldwide each year [1]. While generally well tolerated, acute adverse reactions occur across a clinical spectrum from mild, self-limited symptoms (eg, flushing and limited urticaria) to severe, life-threatening physiology (eg, hypotension, bronchospasm, and/or airway edema). The American College of Radiology (ACR) estimates allergic-like reactions to modern iodinated contrast media at approximately 0.6% overall and 0.04% severe, while recent ESUR guidance cites immediate hypersensitivity reaction frequencies of approximately 0.15%-0.69%, with severe reactions reported in approximately 0.005%-0.013% [2,3]. Regardless of mechanism, early recognition and prompt treatment, particularly epinephrine for anaphylaxis, are consistently emphasized in radiology and emergency medicine guidance [2,4,5].

In parallel with these longstanding safety principles, radiology practice has undergone substantial operational transformation driven by staffing constraints, multisite outpatient expansion, and increasing adoption of telemedicine-enabled workflows. Regulatory policy has evolved alongside these changes. Most notably, the Centers for Medicare and Medicaid Services (CMS) finalized its CY 2026 Physician Fee Schedule allowing direct supervision through real-time 2-way audio-video communication (“virtual direct supervision”) indefinitely for specified services, including diagnostic imaging [6,7].

Professional societies have also addressed this shift. The ACR Drugs and Contrast Media Committee recognizes virtual supervision as a potential model when qualified on-site personnel are able to recognize adverse reactions, administer medications such as epinephrine, perform interventions under standing orders, and activate emergency response systems [8,9]. These regulatory dynamics, paired with technology maturation, have facilitated broader adoption of telemedicine-enabled clinical oversight models in outpatient imaging.

In many contemporary virtual supervision models, the supervising physician is connected to imaging centers via a persistent audiovisual presence at the start of the clinical day, with emergency alerts serving to immediately route the physician’s attention to a specific imaging center, scanner, or room rather than initiating first contact.

Despite these developments, limited literature exists describing high-acuity emergencies that are directly witnessed and managed by physicians who are not physically present at the imaging site. Beger et al. [10] recently reported a case of cardiac arrest recognized via video call, highlighting the unique challenges of remotely observed emergencies [10]. As virtual oversight becomes more common in radiology, similar scenarios, including severe contrast reactions, may increasingly occur under tele-supervised or “virtually supervised” conditions.

Here, we describe a severe immediate ICM-associated anaphylaxis with upper-airway angioedema that was managed initially via a telemedicine-enabled supervision platform with remote physician guidance and on-site technologist execution. We present the clinical course and discuss implications for preparedness, technology reliability, and training in distributed imaging environments.

## Case presentation

A 63-year-old woman with no known prior contrast reaction presented for outpatient CT pelvis with iodinated contrast at a community imaging center. Past medical history included hypertension, diabetes mellitus, coronary artery disease, and osteoarthritis. She denied asthma and reported no known drug allergies. Per routine workflow, 100 mL of a nonionic, low-osmolality iodinated contrast agent (iohexol) was administered via power injector at 11:07 AM.

As part of routine workflow, a supervising physician was already connected to the imaging center at the start of the day through a persistent real-time audiovisual supervision session for contrast administration.

Within approximately 2 minutes of injection, the patient reported pruritus and developed visible urticaria over the torso. The technologist stopped the scan and initiated the site’s emergency response protocol using the imaging center’s telemedicine-enabled supervision platform (Tether Supervision; Houston, TX).

At 11:10 AM, a radiologic technologist activated the platform’s emergency alert, which immediately notified and routed the remote supervising physician to the correct imaging center. Live video communication was established with the supervising physician within approximately 20 seconds. Under video assessment, the patient appeared pale and confused with diffuse urticaria on the upper extremities. The technologist obtained vital signs and performed a sternal rub. A blood pressure of 70/47 mmHg was recorded and the patient's heart rate was 111 beats per minute. Pulse oximetry displayed an SpO_2_ of 70%-80% with a poor waveform, and respirations appeared labored with profound tachypnea at approximately 30 breaths per minute.

The clinical constellation of acute urticaria plus hypotension and respiratory compromise met accepted criteria for anaphylaxis. Given the rapid progression, hypotension, hypoxemia, and concern for upper-airway involvement, the remote physician advised high-flow oxygen via non-rebreather mask at 15 L/min and directed administration of 0.3 mg of intramuscular epinephrine via autoinjector into the anterolateral thigh at 11:11 AM. The physician also guided airway positioning maneuvers (jaw-thrust) in response to probable upper-airway obstruction and instructed the patient to be placed in Trendelenburg position. At 11:12 AM, emergency medical services were requested.

Over the ensuing minutes, oxygen saturation improved to 93% SpO_2_. Under remote direction, a nasopharyngeal airway was placed. At 11:15 AM, a second dose of 0.3 mg of intramuscular epinephrine via autoinjector was administered due to persistent hypotension and ongoing respiratory compromise. Shortly thereafter, systolic blood pressure improved to 90 mmHg. Given persistent tachycardia, an IV crystalloid bolus was being prepared. Closed-loop communication techniques were used to ensure accuracy and confirm completion of tasks.

Emergency medical services arrived at approximately 11:18 AM, and a brief handoff was provided via the technologist’s device. EMS personnel noted persistent facial flushing and findings concerning for upper-airway edema (including tongue enlargement and muffled voice) while the patient remained able to phonate. EMS initiated an IV crystalloid infusion and administered 125 mg of IV methylprednisolone. Given improving oxygenation with the nasopharyngeal airway and supplemental oxygen, the patient was transported without intubation from the imaging center at 11:22 AM. The remote physician remained connected through departure to support patient care coordination.

At approximately 11:30 AM, on arrival to the emergency department, the patient underwent endotracheal intubation for airway protection. Direct visualization during intubation confirmed suspected progressive laryngeal edema. The patient was subsequently admitted to the ICU for ventilatory support. By the following morning, facial and airway swelling had substantially improved; the patient was extubated approximately 24 hours after intubation and maintained stable hemodynamics without vasopressors. She was discharged home 2 days later with avoidance instructions for iodinated contrast and an epinephrine auto-injector as an individualized precaution pending specialist evaluation. Allergy/immunology follow-up was arranged for further assessment and consideration of confirmatory testing. A timeline of the major clinical events and corresponding telemedicine platform activities is shown in Table 1, illustrating both patient management and the sequence of remote physician engagement during the emergency.

**Table 1:** Timeline of clinical events and interventions.

Time	Clinical event	Telemedicine workflow
Start of day	Patient not yet present	Supervising physician connected via persistent audiovisual supervision session (Tether Supervision)
11:07 AM	Iohexol administered	Physician already connected and available for direct supervision
∼11:09 AM	Pruritus and diffuse urticaria develop; scan stopped	Technologist recognizes reaction
11:10:35 AM	Emergency response initiated	Emergency alert activated and alarm on physician’s HIPAA-compliant computer platform.
∼11:10:55 AM	Initial patient assessment	Physician speaking with on-site team and patient (∼20 seconds after alert)
11:11 AM	First 0.3 mg IM epinephrine; oxygen initiated	Physician remotely assesses patient; directs treatment and airway positioning
11:12 AM	EMS requested	Ongoing physician supervision
∼11:13 AM	Oxygen saturation improves; nasopharyngeal airway placed	Continued video assessment and closed-loop communication
11:15 AM	Second 0.3 mg IM epinephrine	Physician reassesses patient; directs repeat dosing
11:18 AM	EMS arrival	Remote physician participates in EMS handoff
11:22 AM	EMS departure	Physician remains connected until transfer completed
∼11:30 AM	Emergency department intubation	—
Hospital Day 2	Extubation	—
Hospital Day 3	Discharge home	Physician follows up with patient for aftercare instructions

## Discussion

This case is consistent with ICM-associated anaphylaxis, with urticaria, distributive shock physiology, hypoxia, and upper-airway angioedema. Although severe ICM-associated anaphylaxis is rare in aggregate, its time-sensitive nature makes preparedness essential, particularly in outpatient settings, where emergency response teams may not be immediately available. Published estimates suggest severe reactions occur in the approximate range of 0.005%-0.04% of all administrations, with variation by agent, definitions, and ascertainment [2,3].

### Medical management considerations

Contrast reaction terminology remains inconsistent across specialties. In contemporary allergy and immunology literature, these reactions are generally classified as immediate hypersensitivity reactions and, when accompanied by airway, breathing, or circulatory compromise, as anaphylaxis. The GA²LEN 2024 consensus report defines anaphylaxis as a serious systemic allergic or hypersensitivity reaction that may progress rapidly and become fatal, with life-threatening anaphylaxis characterized by airway, breathing, and/or cardiovascular compromise [11]. In this case, the patient developed abrupt urticaria, hypotension, hypoxemia, altered responsiveness, and suspected upper-airway involvement immediately following iodinated contrast exposure, findings that meet clinical criteria for iodinated contrast media-associated anaphylaxis.

However, the pathophysiology of iodinated contrast media reactions is heterogeneous and not always IgE-mediated. Immediate hypersensitivity reactions to nonionic contrast media may occur through IgE-dependent mechanisms, complement-dependent pathways, direct membrane effects, non-IgE-dependent mast-cell activation, or other mechanisms; clinically, IgE-mediated and non-IgE-mediated anaphylaxis can be indistinguishable at the bedside [3]. This mechanistic uncertainty partly explains persistent differences in terminology. The ACR Manual continues to classify acute contrast reactions pragmatically as “allergic-like” vs “physiologic,” whereas ESUR 2025 uses “hypersensitivity reactions,” “immediate hypersensitivity reactions,” and “nonimmediate hypersensitivity reactions,” while acknowledging that the ACR classification remains practical for radiology employees because it clearly distinguishes reactions requiring action from those that do not [2,3]. Similarly, recent CAR/CSACI practice guidance uses “hypersensitivity reaction” as the umbrella term, separates physiologic/chemotoxic reactions from hypersensitivity reactions, and describes “non–IgE-mediated anaphylaxis” as the current term for reactions formerly called “anaphylactoid” [12]. Thus, in this report we use “iodinated contrast media-associated anaphylaxis” for the clinical diagnosis, while recognizing that the same event may be categorized operationally in radiology as a severe immediate allergic-like contrast reaction.

Regardless of nomenclature or mechanism, epinephrine is the central first-line therapy for severe contrast-associated anaphylaxis. Epinephrine addresses the major lethal physiology of anaphylaxis through α-adrenergic vasoconstriction and reduction of mucosal edema, β1-mediated cardiac support, β2-mediated bronchodilation, and reduced mediator release [13]. ESUR 2025 describes intramuscular adrenaline/epinephrine as the most important drug for severe immediate hypersensitivity reactions and emphasizes supportive oxygen and volume expansion [3]. The 2020 Joint Task Force practice parameter similarly states that epinephrine is first-line pharmacotherapy for anaphylaxis and that antihistamines or glucocorticoids are not reliable interventions to prevent biphasic anaphylaxis [13].

Epinephrine dosing for adult contrast-associated anaphylaxis is an area where radiology contrast-reaction protocols and general anaphylaxis guidance do not fully align (Table 2). Many general anaphylaxis guidelines recommend 0.01 mg/kg intramuscular epinephrine up to 0.5 mg in adults, and ESUR 2025 notes that most adult anaphylaxis guidelines recommend 0.5 mg IM [3,13]. In contrast, ACR adult contrast-reaction tables list 0.3 mg IM epinephrine or a fixed 0.3 mg adult epinephrine auto-injector dose, with repeat dosing every 5-15 min, including for severe bronchospasm, laryngeal edema, and hypotension with suspected anaphylactic physiology [2]. Therefore, the 0.3 mg IM doses in this case should be interpreted as consistent with common ACR-style radiology contrast-reaction protocols, while lower than the 0.5 mg adult dose emphasized in many general anaphylaxis guidelines.

**Table 2:** Comparison of adult intramuscular epinephrine recommendations for anaphylaxis.

Guideline	Adult IM epinephrine dose	Repeat dosing	Comments
ACR Manual on Contrast Media (2024)	0.3 mg IM	Every 5-15 min as needed	Contrast-specific guidance; consistent with adult epinephrine auto-injector dosing
ESUR Contrast Media Safety Committee (2025)	Up to 0.5 mg IM	Every 5-15 min as needed	Notes that most adult anaphylaxis guidelines recommend a 0.5 mg dose
World Allergy Organization (WAO, 2020)	0.01 mg/kg (maximum 0.5 mg)	Every 5-15 min as clinically indicated	Prompt IM injection into the anterolateral thigh is first-line therapy
Joint Task Force Practice Parameter (AAAAI/ACAAI, 2020)	0.01 mg/kg (maximum 0.5 mg)	Every 5-15 min if symptoms persist	Emphasizes early IM epinephrine; do not delay for adjunctive therapies
Resuscitation Council UK (2021)	500 µg IM (0.5 mg)	Every 5 min if required	Repeat dosing based on ongoing airway, breathing, or circulatory compromise

Recommendations summarized from references 2, 3, 4, 13, and 24. The ACR Manual on Contrast Media recommends a fixed 0.3 mg intramuscular dose for severe contrast reactions, whereas most general anaphylaxis guidelines recommend 0.01 mg/kg up to a maximum adult dose of 0.5 mg.

AAAAI, American Academy of Allergy, Asthma, and Immunology; ACAAI, American College of Allergy, Asthma, and Immunology; ACR, American College of Radiology; ESUR, European Society of Urogenital Radiology; IM, intramuscular; WAO, World Allergy Organization.

The use of epinephrine auto-injectors may be particularly relevant in outpatient imaging centers because severe contrast reactions are rare, high-stress events, and radiology-specific data show that epinephrine dosing, concentration, route, and equipment errors are common. In a North American survey of 253 radiologists, 91% selected epinephrine as the key initial medication for a severe contrast reaction, but no radiologist gave the ideal response; only 41% gave an acceptable route/concentration/dose, 17% gave an overdose, and only 11% knew the concentration of epinephrine available in their kit or crash cart and the equipment required to administer it [14]. In a high-fidelity bi-institutional simulation of severe allergic-like contrast reactions, 58% of radiology residents, fellows, and faculty committed an epinephrine administration error, including IV epinephrine without appropriate flush/fluids, overdose, and IV administration of 1 mg/mL epinephrine [15]. After a sentinel event involving IV epinephrine overdose in a radiology department, Nandwana et al. [16]. found that only 52% of tested radiology personnel correctly identified the IM epinephrine dose and only 29% knew the IV dose and rate 4 In another surprise mock severe contrast reaction study, epinephrine administration errors occurred in 61%-63% of scenarios and were not predicted by postgraduate year or time since recent training; IM administration was faster than IV administration [17].

Auto-injectors directly address several of these human-factors risks. In a simulation study comparing manual IM epinephrine draw-up/injection with auto-injector use for contrast reactions, manual delivery was significantly slower than auto-injector delivery, with mean administration times of 108.8 vs 38.7 seconds, and the manual group had more errors, 11 vs 1 [18]. Participants also reported greater comfort with auto-injectors. A CADTH evidence review found no direct real-world comparative clinical-effectiveness studies of auto-injectors vs manual epinephrine in actual anaphylaxis events, but concluded that 2 radiology simulation studies showed quicker administration and fewer errors with epinephrine auto-injectors [19]. Commonly used U.S. adult epinephrine auto-injectors deliver 0.3 mg rather than 0.5 mg, and repeat dosing with a second device may be necessary when symptoms persist or worsen [20,21].

The medicolegal literature also supports emphasizing timely recognition and treatment rather than focusing narrowly on the nominal first dose. In a U.S. legal database analysis of 151 contrast-related malpractice cases, anaphylactic reaction after contrast administration was the most common alleged complication, accounting for 30% of cases; among those anaphylaxis cases, 93% involved allegations of failure to diagnose developing anaphylaxis or failure to treat the anaphylactic reaction [22]. A separate analysis of anaphylaxis-related malpractice lawsuits found that 40% involved IV contrast as the trigger, 40% cited delayed recognition or treatment, and 17% involved inappropriate IV epinephrine dosing [23].

In the present case, airway compromise was the dominant immediate concern. The patient had profound hypoxemia with labored respirations, suspected upper-airway involvement, and later EMS documentation of tongue enlargement and muffled voice, followed by emergency-department intubation for airway protection. Initial management therefore appropriately prioritized epinephrine, high-flow oxygen, airway positioning, jaw thrust, and nasopharyngeal airway placement. IV fluids are recommended for anaphylaxis-associated hypotension, and both ACR and ESUR include volume expansion in severe reaction algorithms [2,3].

Corticosteroids should also be framed as adjunctive rather than primary therapy in outpatient imaging emergencies. Glucocorticoids have a delayed onset of action and are not life-saving in the initial phase of anaphylaxis; their proposed role in preventing protracted or biphasic symptoms remains uncertain [5,13]. The 2020 Joint Task Force practice parameter states that antihistamines and glucocorticoids are not reliable interventions to prevent biphasic anaphylaxis [13]. Resuscitation Council UK guidance similarly emphasizes IM adrenaline/epinephrine for airway, breathing, or circulation problems and notes that steroids are no longer recommended for routine initial treatment of anaphylaxis [24].

### Telemedicine-specific considerations

This case also illustrates operational considerations that may influence outcomes when emergencies are remotely treated.

### Time-to-expert engagement

The supervising physician was visually engaged with the patient within approximately 20 seconds. Importantly, the physician was not contacted de novo at the time of the reaction but was already connected and engaged in active virtual supervision and familiar with the site and staff of the imaging center. The emergency alert function on the application served to escalate and localize the physician’s attention to the affected scanner rather than to establish physician connectivity or availability.

### On-site readiness

Before this event, the responding technologist had completed a physician-led contrast-reaction management training program provided by Tether Supervision and the imaging center. The training addressed recognition of contrast reactions, emergency escalation, and performance of physician-directed interventions during an acute reaction. Effective early management depended on on-site personnel’s ability to recognize anaphylaxis and initiate physician-ordered treatment, including intramuscular epinephrine, oxygen, IV fluids when indicated, airway positioning, and basic airway devices. This underscores that remote supervision does not substitute for on-site clinical competence.

### Communication and handoff reliability

Remote participation may improve information continuity and reduce ambiguity during EMS handoff, but this benefit depends on clear role delineation and structured communication. Prior reports of remotely witnessed emergencies emphasize similar themes: remote observers can recognize emergencies and guide early steps, but must contend with limited physical agency and reliance on intermediaries [10,25].

### Policy context and durability

Federal Medicare payment and supervision rules and state scope-of-practice laws govern distinct components of virtual direct supervision. Under the CY 2026 Physician Fee Schedule, CMS permits the presence required for direct supervision to be furnished through real-time interactive audio-video communication for many diagnostic tests. This federal rule does not itself displace state licensure or scope-of-practice requirements, and state law and facility policy may impose additional or more restrictive conditions. Because virtual contrast supervision remains an evolving model, some jurisdictions may not expressly address its use. A key variable is the required credentials of on-site personnel and the interventions they are authorized to perform at the supervising physician’s direction.

This case suggests that a radiologic technologist with dedicated contrast-reaction training can be a valuable member of the on-site response team. The technologist’s rapid recognition of deterioration, execution of physician-directed interventions, and closed-loop communication were central to the early response. Where authorized by state law and facility policy, role-specific training and documented competency may strengthen the safety of virtual supervision.

### Quality and safety implications

Remotely witnessed emergencies introduce distinct operational risks, including communication latency, reliance on intermediaries, and handoff complexity, highlighting the need for rigorous protocols and system-level redundancy, as illustrated by prior reports of emergencies recognized via video communication [10,25].

### Limitations

This report is limited by its single-case design and reliance on clinical documentation and timestamped platform events, which may not capture all actions with perfect precision. No acute serum tryptase or later baseline tryptase was obtained, which limits diagnostic confirmation and follow-up risk assessment [5]. Additionally, the contribution of telemedicine involvement to the patient’s favorable outcome cannot be determined from a single case.

## Conclusion

This case describes ICM-associated anaphylaxis with upper-airway angioedema that was initially managed through a telemedicine-enabled supervision platform combining on-site technologist action with real-time remote physician guidance. To our knowledge, this represents the first detailed description of such a high-acuity contrast reaction managed through a telemedicine-enabled supervision model.

The observed course suggests that, with adequate preparedness and training, virtually supervised emergency response may support timely assessment, recognition, and coordination of care. At the same time, the case highlights the continued importance of robust on-site anaphylaxis preparedness, including standardized epinephrine protocols, airway equipment, IV fluid capability, reliable audiovisual connectivity, and simulation-based training as distributed imaging care models continue to expand.

Future work should focus on outcomes from larger numbers of tele-supervised contrast reactions and on defining best practices for escalation, documentation, communication, and EMS handoff in distributed imaging environments.

## Declaration of generative AI and AI-assisted technologies in the manuscript preparation process

During the preparation of this work, the author(s) used ChatGPT to proofread the article for grammatical errors. After using this tool/service, the author(s) reviewed and edited the content as needed and take(s) full responsibility for the content of the published article.

## Ethics approval

This case report describes routine clinical care and does not involve a research study requiring institutional ethics committee approval. Ethical standards were observed in accordance with local institutional policies.

## Availability of data and materials

No datasets were generated or analyzed during the current study.

## Authors’ contributions

Ravi V. Soni collected clinical information, participated in patient care, and drafted the initial manuscript. Samuel B. Beger conceived the case report, supervised the project, and critically revised the manuscript for important intellectual content. William Ji provided remote supervision during the event, contributed to the interpretation of the telemedicine aspects, and assisted with manuscript revisions. All authors read and approved of the final manuscript.

## Patient consent

Written informed consent was obtained from the patient for publication of this case report and any accompanying images. A copy of the written consent is available for review by the Editor-in-Chief of this journal.
